# The association between double-lumen tube versus bronchial blocker and postoperative pulmonary complications in patients after lung cancer surgery

**DOI:** 10.3389/fonc.2022.1011849

**Published:** 2022-09-27

**Authors:** Wei Liu, Fan Jin, He-Mei Wang, Fang-Fang Yong, Zhen Wu, Hui-Qun Jia

**Affiliations:** ^1^ Department of Anesthesiology, The Forth hospital of Hebei Medical University, Shijiazhuang, China; ^2^ Department of Anesthesiology, Zhuji People’s Hospital, Shaoxing, China

**Keywords:** double-lumen tube, bronchial blocker, lung isolation, complications postoperative, lung cancer, one-lung ventilation

## Abstract

**Background:**

Both double-lumen tube (DLT) and bronchial blocker (BB) are used for lung isolation in patients undergoing lung cancer surgery. However, the effects of different devices for lung isolation remain inconclusive. Present study was designed to investigate the association between the choice of the two devices and postoperative pulmonary complications (PPCs) in patients with lung cancer.

**Methods:**

In this retrospective cohort study, patients who underwent lung cancer surgery between January 1, 2020 and October 31, 2020 were screened. Patients were divided into two groups according to different devices for lung isolation: DLT group and BB group. Primary outcome was the incidence of a composite of PPCs during postoperative in-hospital stay.

**Results:**

A total of 1721 were enrolled for analysis, of them, 868 received DLT and 853 BB. A composite of PPCs was less common in patients with BB (25.1%, [214/853]) than those received DLT (37.9% [329/868] OR 0.582 95% CI 0.461-0.735 P < 0.001). Respiratory infection was less common in BB group (14.4%, [123/853]) than DLT group (30.3%, [263/868], P<0.001). The incidence of non-PPCs complications was not statistically significant between the 2 groups.

**Conclusions:**

For patients undergoing surgery for lung cancer, the use of BB for lung isolation was associated with a reduced risk of PPCs when compared with DLT.

## Introduction

Lung cancer is the second most commonly diagnosed cancer and the leading cause of cancer death worldwide in 2020 (1). Surgical resection is mostly the treatment of early-stage peripheral lung cancer. Because of surgical trauma and perioperative factors, such as one-lung ventilation (OLV) and ischemia-reperfusion/hypoxia-reoxygenation injury, postoperative pulmonary complications (PPCs) are still the major cause of postoperative morbidity and mortality which had tremendous influence on patients’ outcome (2). The most common PPCs after lung resection include respiratory infection, pleural effusion, aelectasis and acute respiratory distress syndrome, with a reported incidence of 30-55% (3–5). PPCs can lead to increase hospitalization expense, prolong hospital stays and even increase perioperative morbidity and mortality (5–7).

Previous studies reported that risk factors for PPCs after lung surgery were patient characteristics, preoperative testing, type of operation and anesthetic management (8, 9). As an indispensable part in anesthetic management for lung cancer, OLV can be achieved using a double-lumen tube (DLT) or bronchial blocker (BB). The DLT is stiffer and bulkier than a standard single-lumen tube (SLT), and there is a risk of tracheobronchial injury (10–12). The BB is inserted inside a SLT previously placed into the trachea, while the blood or secretions from the operative lung may expose the contralateral lung to contamination. Several studies of different airway devices (DLT or BB) for lung isolation on PPCs showed different results (13, 14). A randomized controlled study showed that implementation of BB has lower incidence of pulmonary infection within 1 week after thoracic surgery than DLT (13). However, a retrospective cohort study revealed that patients in BB group had high risks of pulmonary infection or respiratory failure in the first postoperative year than those in DIL group (14).

Evidence regarding airway devices for lung isolation and PPCs is likewise mixed with the types of the surgeries and postoperative follow-up time. The effect of DLT versus BB on PPCs after lung resection surgery remain uncertain. Therefore, the current study was designed to assess the impact of the different airway devices on PPCs during postoperative hospitalization in patients after lung cancer surgery.

## Methods

### Study design

This was a retrospective single-center cohort study which was approved by Ethics Committee of The Forth Hospital of Hebei Medical University (2022KS009). Because all variables were collected *via* electronic medical record system, the local Ethics Committee agreed to exempt the written informed consent. The trial was registered prior to patient enrollment at Chinese Clinical Trial Registry (chiCTR2200060037).

### Patients

Adult patients (age≥ 18-year-old) who underwent lung resection surgery between January 1, 2020 and October 31, 2020 in The Forth Hospital of Hebei Medical University were screened. Patients who met any of the following criteria were excluded:

- benign tumor (based on pathology report)
- biopsy procedure
- bilateral pulmonary resection
- preoperative respiratory infection confirmed by computed tomography (CT)
- planned to receive reoperation or postoperative mechanical ventilation
- incomplete data collected from the electronic medical record system or anesthesia record system.

### Anesthesia and perioperative care

In operating room, all patients were monitored by electrocardiogram, pulse oxygen saturation (SpO_2_), and non-invasive blood pressure, end-tidal carbon dioxide (ETCO_2_), bispectral index (BIS), and nasopharyngeal temperature. Continuous blood pressure was measured by radial puncture under local anesthesia before the induction of anesthesia.

Anesthesia was induced by intravenous administration of propofol and/or etomidate, sufentanil, and rocuronium or cis-atracurium. DLT (Teleflex Medical) or BB (Hangzhou Tappa Medical Technology CO., Hangzhou, China) was used for lung isolation, which was chosen according to the status of patients, the experience of anesthetist and the preferences of surgeon. After intubation, fiberoptic bronchoscopy was applied to ensure the DLT/BB appropriate position. Sevoflurane inhalation or propofol infusion and opioids (such as remifentanil or sufentanil) were used for anesthesia maintenance. Muscular relaxants (i.e., rocuronium and cis-atracurium) were administered to maintain surgical field condition. Regional nerve block (i.e., epidural anesthesia, paravertebral nerve block, intercostal nerve block and erector spinae plane block) could be used for perioperative analgesia. Patient-controlled intravenous analgesia was provided for postoperative pain management. The aim of anesthesia was to maintain: BIS 40-50, blood pressure fluctuation within 20% baseline value, and temperature 36-37°C.

After endotracheal intubation, volume control mode was used for ventilation. Tidal volume (TV) was set at 6-8 mL/Kg of ideal body weight during two lung ventilation and 5-6 mL/Kg during OLA, and positive end-expiratory pressure (PEEP) was used in necessary and set at 5-10 cmH_2_O. Respiratory rate was adjusted to maintain a target P_ET_CO_2_ of 35-45 mmHg. Inspired fraction of oxygen (FiO_2_) was set at 1.0 during induction to assure oxygenation conservation for intubation, and then initially decreased to 0.6-0.8 to keep SpO_2_≥ 92% during mechanical ventilation. When hypoxemia occurred during OLV, the intervention included an increase in FiO_2_ followed by a recruitment maneuver and escalation of PEEP to the ventilated lung. If this failed to correct the SpO_2_ to a perceived acceptable level, then low-level continuous positive airway pressure (CPAP) might be applied to the operative lung.

Fluid infusion was given in line with routine practice. Crystalloid was administrated at a rate of 4–6 mL/kg/h. Colloids or allogenic blood product was administrated according to patient’s condition and the discretion of attending anesthesiologists.

At the end of anesthesia, patients were extubated as soon as appropriate and muscular relaxant was antagonized by neostigmine 0.02–0.04 mg/kg and atropine 0.01–0.02 mg/kg when regarded as clinically necessary by the attending anesthesiologist.

### Date collection

Date was collected retrospectively using the electronic medical record system or anesthesia record system. Preoperative data included demographic characteristics (such as sex, age, height and weight), comorbidity, pulmonary function, and arterial blood gas analysis. Intraoperative data included type and duration of surgery, surgical lobe, type and duration of anesthesia, fluid infusion, blood transfusion, the use of neostigmine, the highest and lowest arterial oxygen partial pressure (PaO_2_). Postoperative data included occurrence of PPCs and non-PPCs complications (i.e., stroke, atrial fibrillation, acute heart failure, cardiac arrest and sepsis), unplanned ICU admission and in-hospital mortality.

### Exposure variables

Exposure variables were different airway devices for lung isolation. To analyze the effect of different airway devices on PPCs in patients after lung cancer surgery, patients were classified into two groups: DLT and BB.

### Primary outcome

Primary outcome was to compare the influence of DLT and BB on PPCs during postoperative in-hospital stay (from the end of operative to hospital discharge) in patients after lung cancer surgery.

PPCs is a syndrome with a predefined composite outcome including respiratory infection, pleural effusion, atelectasis, respiratory failure, bronchospasm, aspiration pneumonitis, and pneumothorax (15).

Respiratory infection was diagnosed if it met with at least one of the following criteria and treated by antibiotics: new or changed sputum, new onset or alteration in lung opacities, temperature≥ 38.3°C and leukocyte count≥ 12,000/μL. Pleural effusion was diagnosed by chest X-ray and the need of medical drainage for treatment. Atelectasis was diagnosed by chest X-ray and the need for medical treatment such as oxygen supply and physical training. Respiratory failure was diagnosed if PaO_2_≤ 60 mmHg or PaCO_2_≥ 45 mmHg on room air or a ratio of PaO_2_/FiO_2_< 300 and requiring oxygen therapy or mechanical ventilation. Pneumothorax was confirmed by X-ray and the need of treatment was also recorded. New onset bronchospasm was diagnosed according to clinical manifestations and the need of bronchodilators for treatment. Given the effect of surgical factors, we considered pleural effusion and pneumothorax to be complications only when they occurred in the contralateral lung (16).

### Secondary outcome

Secondary outcome was the incidence of non-PPCs complications such as stroke, atrial fibrillation, acute heart failure, cardiac arrest and sepsis. We also collected unplanned ICU admission and in-hospital mortality as secondary outcome.

### Statistical analysis

#### Outcome analysis

Normal distribution was tested by Histograms and Q-Q plots. Continuous data with normality were presented as mean ± standard deviation (SD) and analysed using Student’s *t* test. If Continuous data were abnormality, Kruskal-Wallis test was used and median (interquartile range, IQR) were presented. Categorical data were expressed as frequency (percentage) and compared by chi-square test, continutity correction or Fisher’s exact test.

For primary outcome analysis, the incidence of PPCs was compared by Chi-square test. Univariate analysis was firstly used to screen potential variables in relation with PPCs. Variables with P< 0.05 were entered into multivariable logistic regression to examine independent risk factors of PPCs. The incidence of non-PPCs complications, unplanned ICU admission and in-hospital mortality was compared by chi-square test, continutity correction or Fisher’s exact test.

All statistical analyses were performed with SPSS 25.0 (SPSS, Inc., Chicago, IL., USA). For all tests, a two-sided P < 0.05 was considered statistically significant.

#### Power analysis

A power analysis was performed using a two-sided Z test with unpooled variance. A sample size of 868 in DLT group and 853 in BB group can provided 99.9% power at an alpha = 0.05 to detect a 12.8% difference in the rate of PPCs, while the incidence of PPCs was 37.9% in DLT group and 25.1% in BB group.

## Results

### Participants

**Figure 1:**
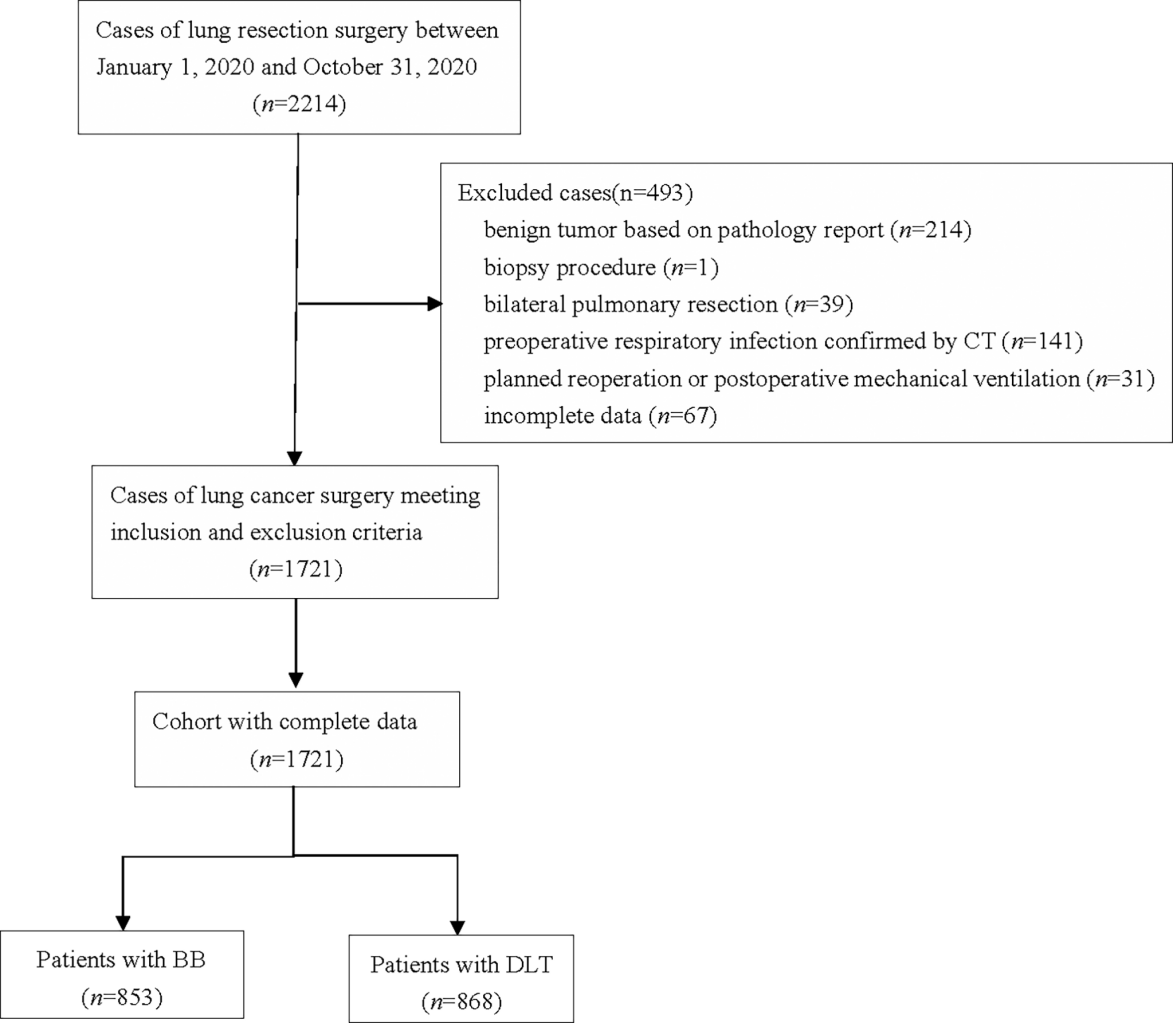
Flowchart of the study.

A total of 2214 patients who underwent lung surgery between January 1, 2020 and October 31, 2020 were screened. Among them, 214 patients were excluded for benign tumor based on pathology report, 1 for biopsy procedure, 39 for bilateral pulmonary resection, 141 for preoperative respiratory infection confirmed by CT, 31 for planned reoperation or postoperative mechanical ventilation, and 67 for incomplete data collected from the electronic medical record system or anesthesia record system. Finally, 1721 patients met inclusion and exclusion criteria were enrolled for analysis. Among the remaining1721 patients, 868 received DLT for lung isolation, and 853 received BB (**Figure 1**).

### Baseline and preoperative/intraoperative data

Compared with patients received DLT, those with BB had lower fraction of ASA classification ≥3, preoperative chemotherapy and lower baseline PaO_2_ (All P values< 0.05), **Table 1**; they received more left lung surgery and more general anesthesia alone (versus combined regional nerve block); they were given more intraoperative fluid, more colloid and allogenic blood transfusion. Duration of surgery in BB group were shorter compared with DLT group (**Table 2**).

**Table 1:** Baseline and preoperative data.

Variables	Total (n = 1721)	DLT (n = 868)	BB (n = 853)	*P*
Age, yr, mean±SD	59.2 ± 9.9	58.7 ± 10.1	59.8 ± 9.6	0.016**^#^**
>65, n (%)	565(32.8)	266 (30.6)	299 (35.1)	0.052^*^
Male, n (%)	779 (45.3)	396 (45.6)	383 (44.9)	0.764^*^
BMI, kg/m², mean±SD	25.2 ± 3.3	25.0 ± 3.3	25.3 ± .3.3	0.074**^#^**
Preoperative comorbidity, n (%)				
Diabetes mellitus	215 (12.5)	101 (11.6)	114 (13.4)	0.278^*^
Hypertension	599 (34.8)	284 (32.7)	315 (36.9)	0.067^*^
Coronary heart disease	147 (8.5)	66 (7.6)	81 (9.5)	0.160^*^
COPD	88 (5.1)	53 (6.1)	35 (4.1)	0.059^*^
Stroke	440 (25.6)	228 (26.3)	212 (24.9)	0.501^*^
Asthma	11 (0.6)	4 (0.5)	7 (0.8)	0.349^*^
ASA classification, n (%)				<0.001^*^
I or II	1485 (86.3)	717 (82.6)	768 (90.0)	
III or IV	236 (13.7)	151 (17.4)	85 (10.0)	
Chronic smoking, n (%)^a^	460 (26.7)	237 (27.3)	223 (26.1)	0.586^*^
History of lung surgery, n (%)	24 (1.4)	10 (1.2)	14 (1.6)	0.387^*^
Preoperative chemotherapy, n (%)	77 (4.5)	52 (6.0)	25 (2.9)	0.002^*^
FEV_1_, %, median (IQR)	101.1 (90.2,112.0) (n=1656)	100.9 (90.3,111.7) (n=834)	101.4 (90.2,112.5) (n=822)	0.625^&^
PaO_2_, mmHg, median (IQR)	85.8 (78.8,92.7) (n=1331)	87.6 (79.9,94.1) (n=702)	83.8 (77.0,90.9) (n=629)	<0.001^&^

DLT, double-lumen tube; BB, bronchial blocker; SD, standard deviation; IQR, interquartile range; BMI, body mass index; COPD, Chronic Obstructive pulmonary disease; ASA, American Society of Anesthesiologists.

FEV_1_, Forced Expiratory Volume in first one second; PaO_2_, arterial oxygen partial pressure.

^a^Chronic smoking was defined as smoking index ≥ 400.

^#^Student’s t test.

^*^Chi-square test.

^&^Kruskal-Wallis test.

**Table 2:** Intraoperative data.

Variables	Total (n = 1721)	DLT (n = 868)	BB (n = 853)	*P*
Surgical procedure, n (%)				0.234^*^
Lobectomy^a^	1693 (98.4)	857 (98.7)	836 (98.0)	
Pneumonectomy	28 (1.6)	11 (1.3)	17 (2.0)	
Surgical site, n (%)				< 0.001^*^
Left	671 (39.0)	284 (32.7)	387 (45.4)	
Right	1050 (61.0)	584 (67.3)	466 (54.6)	
Surgical approach, n (%)				0.337^*^
VATS	1560 (90.6)	781 (90.0)	779 (91.3)	
Thoracotomy	161 (9.4)	87 (10.0)	74 (8.7)	
Anesthesia type, n (%)				<0.001^*^
GA only	405 (23.5)	110 (12.7)	295 (34.6)	
GA + regional nerve block^b^	1316 (76.5)	758 (87.3)	558 (65.4)	
Arterial blood gas analysis				
Highest PaO2, mmHg, median (IQR)	273.2 (157.8,400.3) (n=1479)	274.9 (161.2,401.0) (n=770)	271.4 (151.0,398.4) (n=709)	0.337^&^
Lowest PaO2, mmHg, median (IQR)	115.4 (81.4,231.2) (n=1389)	119.5 (83.9,213.8) (n=684)	111.8 (79.4,242.4) (n=705)	0.444^&^
Intraoperative fluid balance				
Total input, mL, median (IQR), mL	1300.0 (1100.0,1600.0)	1250.0 (1050.0,1550.0)	1300.0 (1150.0,1600.0)	<0.001^&^
Use of colloid^c^, n (%)	1276 (74.1)	557 (64.2)	719 (84.3)	<0.001^*^
Allogenic blood transfusion, n (%)	48 (2.8)	14 (1.6)	34 (4.0)	0.003^*^
Use of neostigmine, n (%)	1404 (81.6)	697 (80.3)	707 (82.9)	0.167^*^
Duration of surgery, h, median (IQR)	2.7 (2.1,3.3)	2.8 (2.1,3.5)	2.5 (2.0,3.2)	<0.001^&^

DLT, double-lumen tube; BB, bronchial blocker; IQR, interquartile range; VATS, Video-assisted Thoracoscopic Surgery; GA, general anesthesia.

PaO_2_, arterial oxygen partial pressure; SpO_2_, Oxygen saturation measured by pulse oximetry.

^a^Including segmentectomy lobectomy, segmentectomy and local excision.

^b^Regional nerve block included epidural anesthesia, paravertebral nerve block, paravertebral nerve block, and intercostal nerve block.

^c^Including hydroxyethyl starch and gelatin.

^*^Chi-square test.

^&^Kruskal-Wallis test.

### Primary outcome

Total incidence of PPCs after lung cancer surgery was 31.6% (543/1721). The top three individual PPCs were respiratory infection (22.4%), pleural effusion (12.7%), and atelectasis (6.9%). The incidence of PPCs was 25.1% (214/853) in BB group which showed significantly lower in comparison with 37.9% (329/868) in DLT group (P<0.001). The incidence of individual complication, except for respiratory infection (P<0.001), was not statistically significant in comparison with DLT group and BB group (**Table 3**).

**Table 3:** Primary and secondary outcome.

Outcome	Total (n = 1721)	DLT (n = 868)	BB (n = 853)	*P*
Overall PPCs, n (%)	543 (31.6)	329 (37.9)	214 (25.1)	<0.001^*^
Incidence of individual complication, n (%)				
Respiratory infection	386 (22.4)	263 (30.3)	123 (14.4)	<0.001^*^
Pleural effusion	219 (12.7)	119 (13.7)	100 (11.7)	0.216^*^
Atelectasis	119 (6.9)	60 (6.9)	59 (6.9)	0.997^*^
Respiratory failure	9 (0.5)	6 (0.7)	3 (0.4)	0.507^$^
Bronchospasm	1 (0.1)	0 (0)	1 (0.1)	0.496^$^
Incidence of individual non-PPCs complication, n (%)				
Stroke	2 (0.1)	1 (0.1)	1 (0.1)	>0.999^$^
Atrial fibrillation	75 (4.4)	41 (4.7)	34 (4.0)	0.454^*^
Acute heart failure	4 (0.2)	2 (0.2)	2 (0.2)	>0.999^$^
Sepsis	3 (0.2)	2(0.2)	1 (0.1)	>0.999^$^
Unplanned ICU admission, n (%)	7(0.4)	4(0.5)	3(0.4)	>0.999^$^
In-hospital mortality, n (%)	2 (0.1)	1 (0.1)	1 (0.1)	>0.999^$^

DLT, double-lumen tube; BB, bronchial blocker; PPCs, postoperative pulmonary complications.

^*^Chi-square test.

^$^Fisher’s exact test.

In univariate analysis, 27 variables were screened as potential factors with PPCs. We included statistically significant variables (with P<0.05) for multivariable logistic regression. Multivariable logistic regression showed that patients in BB group had a significantly lower risk of PPCs (OR 0.582, 95% CI 0.461-0.735, P<0.001) after adjustment for the above confounders. In addition, multivariable logistic regression demonstrated that airway device, chronic smoking, ASA classification, preoperative chemotherapy and duration of surgery were independent risk factors of PPCs. (**Table 4**)

**Table 4:** Univariate and multivariate analysis of factors independently associated with PPCs.

Variables	Univariate		Multivariate	
	OR (95%CI)	*P*	OR (95%CI)	*P*
Airway device (BB versus DLT)	0.552 (0.449-0.679)	<0.001	0.582 (0.461-0.735)	<0.001
Age (≥65 versus <65)	1.190 (0.960-1.474)	0.112		
Male (yes versus no)	1.715 (1.397-2.105)	<0.001		
BMI (per kg/m² increase)	1.016 (0.986-1.047)	0.306		
Diabetes mellitus (yes versus no)	1.393 (1.036-1.873)	0.028		
Hypertension (yes versus no)	1.120 (0.906-1.385)	0.293		
COPD (yes versus no)	2.266 (1.473-3.487)	<0.001		
Stroke (yes versus no)	1.317 (1.048-1.654)	0.018		
Coronary heart disease (yes versus no)	0.950 (0.659-1.371)	0.786		
Asthma (yes versus no)	0.479 (0.103-2.224)	0.347		
Chronic smoking^a^ (yes versus no)	1.948(1.560-2.433)	<0.001	1.375 (1.000-1.892)	0.050
ASA classification (III/IV versus I/II)	2.030 (1.535-2.684)	<0.001	1.652 (1.204-2.265)	0.002
Preoperative history of lung surgery (yes versus no)	1.303 (0.567-2.997)	0.533		
Preoperative chemotherapy (yes versus no)	4.580 (2.823-7.430)	<0.001	2.177 (1.226-3.867)	0.008
Preoperative FEV_1_ (per % increase)	0.986 (0.981-0.992)	<0.001		
Preoperative PaO_2_ (per % increase)	0.999 (0.993-1.006)	0.866		
Surgery procedure	1.408 (0.655-3.027)	0.381		
(Pneumonectomy versus lobectomy^c^)				
Surgical site (right versus left)	1.096 (0.889-1.351)	0.389		
Surgical approach (Thoracotomy versus VATS)	2.467 (1.778-3.423)	<0.001		
Anesthesia type (GA versus GA+RA)	1.220 (0.955-1.558)	0.112		
Highest PaO_2_ (per mmHg increase)	1.000 (0.999-1.001)	0.785		
Lowest PaO_2_ (per mmHg increase)	1.000 (0.999-1.001)	0.900		
Intraoperative total input (per ml increase)	1001 (1.000-1.001)	<0.001		
Use of neostigmine (yes versus no)	0.710 (0.551-0.916)	0.008		
Use of colloid (yes versus no)	1.270 (1.001-1.611)	0.049		
Intraoperative allogenic blood transfusion (yes versus no)	2.034 (1.144-3.618)	0.016		
Duration of surgery (per h increase)	1.458 (1.329-1.598)	<0.001	1.294 (1.139-1.469)	<0.001

PPCs, postoperative pulmonary complications; OR, odds ratio; CI, confidence interval; DLT, double-lumen tube; BB, bronchial blocker; BMI, body mass index; COPD, Chronic Obstructive pulmonary disease; ASA, American Society of Anesthesiologists.

FEV_1_, Forced Expiratory Volume in first one second.

VATS, Video-assisted Thoracoscopic Surgery.

GA, general anesthesia.

RA, regional anesthesia.

PaO_2_, arterial oxygen partial pressure.

^a^Chronic smoking was defined as smoking index ≥ 400.

^c^Including segmentectomy lobectomy, segmentectomy and local excision.

### Secondary outcome

The incidence of non-PPCs complications was not statistically significant in comparison with DLT group and BB group. There were no significant differences in unplanned ICU admission and in-hospital mortality between DLT group and BB group. (**Table 3**)

## Discussion

Results of this retrospective cohort study showed that, for patients undergoing lung cancer surgery, the use of BB for lung isolation was associated with a reduced risk of PPCs when compared with DLT.

The practice of lung surgery, especially video-assisted thoracoscopic surgery (VATS) depend on the ability of anesthesiologist to collapse lung and selectively ventilation the dependent lung. A collapsed lung could provide a quiet operation field for surgeon, which were achieved by either obstructing a bronchus with a BB or by endobronchial intubation with a DLT. Both devices for lung isolation (DLT versus BB) have their advantages and disadvantages (13, 17–20). For most lung cancer surgery, however, either method may be safe and effective (21). In clinical practice, the choice between the two airway devices is usually made according to the status of patients, the experience of anesthetist and the preferences of surgeon. A survey conducted more than a decade ago showed that 17.8% anesthesiologists regularly used BB for lung isolation (22). But with the popularization of fiberoptic bronchoscope and the progress of thoracic anesthesia, more and more anesthesiologists choose BB for lung isolation. In our result, the utilization rate of BB for lung cancer surgery was 49.6% (853/1721).

Our study adopted the same composite of PPCs in line with ARISCAT study (15, 23). The overall incidence of PPCs was about 31.6%, and respiratory infection was the most common PPCs in our study, with an incidence of 22.4%. This is consistent with what has been found in previous study (24, 25). The use of BB for lung isolation reduced respiratory infection by 15.9%, and the difference was statistically significant. There were no differences in other individual component of PPCs between BB and DLT. Similarly, we found no significant difference in non-PPCs complication, unplanned ICU admission and in-hospital mortality between the two groups.

In the present study, a reduced risk of PPCs was observed in patients with BB than in those with DLT. The result might be attributed to the difference in the nature of the two device and intraoperative management. Firstly, the BB is placed *via* a SLT, while the DLT is inserted directly into bronchus. BBs are easier to initially place than a DLT, which are inserted into the target bronchus under the guidance of fiberoptic bronchoscope. However, DLTs are inserted to the target lung by blind intubation at first. In addition, the DLT is stiffer and bulkier, and the distal end of the DLT lacks a bevel, which may result in more difficulty with passage through the vocal cords. It has been reported that a DLT is associated with a high incidence of sore throat, hoarseness and airway injury (20, 26). Secondly, when tidal volume is consistent during OLA, the airway pressure of patients with BB is lower than that of DLT because the inner diameter of the SLA is thicker (13, 17). It is widely accepted that excessive airway pressure will increase the risk of ventilator-associate lung injury and PPCs (27–29). Finally, anesthesia management for OLA between BB and DLT was different. The cuff of BB was inflated before pleural opening, which was deflated after pleural closing, and the BB was withdrawn from the bronchus. The DLT continued to stimulate the bronchus from intubation until extubation, even the cuff of DLT was deflated after pleural closing. Prolonged stimulation of the bronchus by DLT may also contribute to the increasing risk of PPCs.

The effects of BB and DLT for lung isolation on PPCs had been evaluated, but with conflicting results (13, 14). In the recent randomized controlled trial, authors reported that implementation of BB for patients with thoracic tuberculosis undergoing thoracic-approach debridement had lower incidence of postoperative complications including hoarseness, pharyngalgia and pulmonary infection within 1 week after surgery when compared with DLT (13). Similarly, we also found that for patients with lung cancer, the use of BB can reduce the risk of respiratory infection and PPCs during postoperative in-hospital stay, while the incidence of other individual PPCs (such as pleural effusion, atelectasis, respiratory failure and bronchospasm) did not differ between groups. On the contrary, a retrospective cohort study showed that for patients undergoing thoracic surgery, the use of BB for lung isolation had an increasing risk of readmission with pulmonary infection and respiratory failure in the first postoperative year (14). Although it was a matched-pairs study controlling for patient age, sex, and year of surgery, the population in the study was in 2000-2012 and the types of surgery included pulmonary resection, other respiratory surgery, oesophageal surgery, cardiac and vascular surgery. In addition, the intraoperative variables such as anesthesia type and fluid balance were not included in the analysis. These might be the main reason for the inconsistency with our results.

In addition of airway device, our data also showed chronic smoking, ASA classification, preoperative chemotherapy and duration of surgery were independent risk factors of PPCs. The result of the present study corresponded with the early studies (30–32).

Our study had several limitations. Firstly, this was a single-center retrospective study, which could not be wholly generalization to other practices and procedures. Secondly, the choice between BB and DLT for lung isolation in the study center is largely based on the preference of anesthesiologists and surgeons, and we could not control the effect of selection bias on the result. Thirdly, although some variables were adjusted for in multivariable regression analysis, we cannot exclude residual imbalance and bias produced by other factors in comparing the results between the two groups. Ventilation parameters such as VT, PEEP, and driving pressure may be associated with PPCs, while these variables were unavailable in retrospective data collection. Finally, occurrence of PPCs after discharge (i.e., within postoperative 30 days) was not collected and this might underestimate the incidence of PPCs.

## Conclusions

Results of this retrospective study show that, for patients undergoing surgery for lung cancer, the use of BB for lung isolation was associated with a reduced risk of PPCs when compared with DLT. Further prospective studies are required to confirm our result.

## Data availability statement

The original contributions presented in the study are included in the article/supplementary material. Further inquiries can be directed to the corresponding author.

## Ethics statement

The study was approved by Ethics Committee of The Forth Hospital of Hebei Medical University. Written informed consent for participation was not required for this study in accordance with the national legislation and the institutional requirements.

## Author contributions

WL: This author helped in data acquisition, data analysis, and manuscript drafting. FJ: This author helped in data acquisition, and data analysis. H-MW: This author helped in concept and design, and data interpretation. F-FY: This author helped in data acquisition. ZW: This author helped in data acquisition. H-QJ: This author helped in concept and design, data analysis, data interpretation, revision of the manuscript, and final approval of submission. All authors contributed to the article and approved the submitted version.

## Conflict of interest

The authors declare that the research was conducted in the absence of any commercial or financial relationships that could be construed as a potential conflict of interest.

## Publisher’s note

All claims expressed in this article are solely those of the authors and do not necessarily represent those of their affiliated organizations, or those of the publisher, the editors and the reviewers. Any product that may be evaluated in this article, or claim that may be made by its manufacturer, is not guaranteed or endorsed by the publisher.

## Abbreviation

DLT,double-lumen tube; BB, bronchial blocker; PPC, spostoperative pulmonary complications; OL, Vone-lung ventilation; SLT, single-lumen tube; SpO_2_, pulse oxygen saturation; ETCO_2_, end-tidal carbon dioxide; BIS, bispectral index; TV, tidal volume; PEEP, positive end-expiratory pressure; PaO_2_, arterial oxygen partial pressure; SD, standard deviationI; QR, interquartile range; VATS, video-assisted thoracoscopic surgery; BMI, body mass index; COPD, Chronic Obstructive pulmonary disease; ASA, American Society of Anesthesiologists; FEV_1_, Forced Expiratory Volume in first one second; GA, general anesthesia; OR, odds ratio; CI, confidence interval.
